# Temporal and textual analysis of social media on collective discourses during the Zika virus pandemic

**DOI:** 10.1186/s12889-020-08923-y

**Published:** 2020-05-29

**Authors:** May Oo Lwin, Jiahui Lu, Anita Sheldenkar, Ysa Marie Cayabyab, Andrew Zi Han Yee, Helen Elizabeth Smith

**Affiliations:** 1 Wee Kim Wee School of Communication and Information, Nanyang Technological University, 31 Nanyang Link, Singapore, 637718 Singapore; 2 School of New Media and Communication, Tianjin University, Tianjin, 300072 China; 3 Humanities, Arts and Social Sciences, Singapore University of Technology and Design, 8 Sompah Road, Singapore, 487372 Singapore; 4 Lee Kong Chian School of Medicine, Nanyang Technological University, 59 Nanyang Drive, Singapore, 636921 Singapore

**Keywords:** Zika, Social media, Facebook, Twitter, Public health, Health communication

## Abstract

**Background:**

While existing studies have investigated the role of social media on health-related communication, little is known about the potential differences between different users groups on different social media platforms in responses to a health event. This study sets out to explore the online discourse of governmental authorities and the public in Singapore during the recent Zika pandemic in 2016.

**Methods:**

Social media data were extracted from Facebook and Twitter using retroactive keyword sourcing of the word “Zika” to search for posts and a location filter of “Singapore”. Government posts, public posts, and replies to these original posts were included in the temporal and textual analysis.

**Results:**

Overall, Facebook contained more government and individual content whereas Twitter had more content from news media accounts. Though the relative volume of Zika content from different data sources paralleled the peaks and troughs of Zika activities across time, discourses from different data sources differed in their temporal patterns, such that the public discourse died down faster than the government discourse after the outbreak was declared. In addition, the content of discourses differed among data sources. While government discourse included factual information of the disease, public discourse contained more elements of care such as worry about the risks to pregnant women, and elements of community such as well-wishes to each other.

**Conclusions:**

Our study demonstrates the temporal and content differences between user groups and social media platforms in social media conversations during the Zika pandemic. It suggests that future research should examine the collective discourse of a health event by investigating social media discourses within varied sources rather than focusing on a singular social media platform and by one particular type of users.

## Background

In recent years, there have been significant changes in the way health-related information are communicated. These changes are mainly due to the increasing use of social media, which provide platforms for the creation and exchange of user-generated content [1]. Through these channels, traditional one-way flow of information has been transformed into multi-directional communication where the content is no longer exclusively controlled by official sources [2, 3]. Studies show that social media’s real-time dialogic feature [4] and ability to virally spread information [5] help enhance the interactions between health organizations and the public [6, 7].

A health-related event that led to a massive surge in communication between health organizations and the general public is the recent Zika pandemic in 2015–2016. Zika is a mosquito-borne virus that can cause mild symptoms such as fever and rash, or long-term risks such as birth defects for newborn babies. On February 12,016, the World Health Organization (WHO) declared the disease as an international public health emergency. As a result, large amounts of information on prevention methods, risk areas, transmission rates, and other related matters were released by health organizations and demanded by the public [8, 9]. Individuals were also communicating amongst themselves, and with health organizations, in an effort to reduce the resulting uncertainty and anxiety [10, 11].

Although the role of social media has been studied by a number of researchers during the Zika pandemic, little is known about how different user groups in the society participated in the Zika discourse on different social media platforms. Existing studies have tended to focus on the content of Zika discourse [12, 13], dissemination of valid and invalid information [8, 14], and the posting of responses such as recommendations, complaints, and suggestions from the general public to health organizations [15]. However, these studies often focused on the use of a singular social media platform, and by one particular type of user (e.g. governments, health organizations, or the individual user). Since social media platforms differ significantly in their characteristics, and different users can have very distinct communication needs, there are potentially intriguing differences in the use of these online channels for communication purposes. Likewise, there is a need to investigate the discourses that amass from the delivery and exchange of Zika-related information between and amongst the public and the governmental authorities. These discourses reflect the collective response to the disease, and thus lends itself to a rich investigation of not only the communication patterns, but also the emotional and behavioral responses occurring during the Zika outbreak.

To advance the understanding of the potential differences between users and social media platforms in response to the Zika pandemic, this study sets out to explore the online discourses of governmental authorities and the public in Singapore. The localized spread of the Zika virus was confirmed by Singapore’s Ministry of Health (MOH) on August 2016, with about 450 people identified as having been infected by the end of that year [16, 17]. Singapore has been honored by the WHO as a role model to manage the Zika crisis due to its transparency and the quick reporting from the government [18]. This presents Singapore the ideal locale of focus for the analysis of Zika-related social media communication. By extracting and analyzing Zika-related social media posts and replies, this study examines the collective discourses from both government agencies and the general public that emerges over time across two different social media platforms, namely Facebook and Twitter. This can provide important insights for future research and for health organizations in future health epidemics.

## Method

### Design & Data Collection

The objective of this study is to monitor social media discourses of the Singapore general public and government agencies by examining posts, responses, and discussions that arose during the Zika outbreak in Singapore. To achieve this, we collected data from two popular text-based social media platforms-Twitter and Facebook- from 1st October 2015 to 31st December 2016. The outbreak period was defined as 7 months pre and post the first reported case of Zika in Singapore which occurred in May 2016.

We collected the data from the Twitter and Facebook standard search application programming interfaces (API). Data from the three relevant government agencies dealing with the Zika outbreak, The Ministry of Health (MOH), National Environment Agency (NEA) and the Health Promotion Board (HPB), were extracted from their official Facebook and Twitter homepages. These data were defined as “government data”. All other data, including posts/tweets published by individual users and news media accounts, they were defined as “public data”. Public data were extracted using retroactive keyword sourcing of the word “Zika” to search for posts/tweets with a geolocation tag indicating as “Singapore” or with explicit mentions of “Singapore”. The location limit was to ensure the extracted data were published within Singapore. For the Facebook platform, in addition to government and public posts, replies to these posts were also examined to explore the discourses toward the original posts. This could not be done on Twitter due to the limited resources and time constraints. The timestamp of the post/reply was collected to explore where within the timeframe of the outbreak the post/reply was located. At the time of data collection, all the data we extracted were publicly accessible.

For the purposes of comparison, posts/tweets tagged as published in Singapore or explicitly mentioning Singapore within the same period containing the keyword “dengue” were also collected. The official number of confirmed Zika cases within Singapore was obtained from the MOH. Search frequency data for the term “Zika” were also collected using Google Trends across the same period in Singapore for reference. Google Trends is a free online tool to study search data for a specific topic [19]. It analyses the relative frequency of a particular search term (e.g., “Zika”) regarding the total search volume in a specified location and time. The data were expressed in the relative search volume (RSV), with the peak volume of a query expressed as 100 and the other values rescaled to the proportion of the peak (i.e., a score of 50 means the popularity is half of the peak volume). We used “Zika” as a search term instead of the “Zika fever” as a disease in Google Trends. This is because Zika shares the same vector and similar symptoms with Dengue, using “Zika fever” as a disease topic as the search query may likely include those searches that are not only Zika but also dengue or other similar symptoms. The search data from Google Trends has been previously used as a surveillance tool to indicate the public interest in a particular topic and predict the spread of the infectious disease [19–21].

### Data analysis

Once the parameters had been set and the data was extracted. Duplicate posts and replies were removed. Each of the data rows was examined individually by platform and by group, and then removed if deemed as duplicate, while also accounting for other identifiers such as the username, date, and timing of post/reply. The data rows for public posts were also identified by its handles based on four categories of accounts: company/community accounts for the official use of a company or a community; other government accounts for government agencies except the MOH, NEA, and HPB; individual accounts for individual use; and news media accounts for the news distribution. The timestamp of the post was converted into epidemiological weeks for comparative purposes. The epidemiological weeks started on Sunday each week, which is defined by the MOH in Singapore.

Following this, the data were further cleaned to allow for textual analysis. Common English stop words from the SMART information retrieval system predefined by the R package “stopword” [22] such as ‘the’ ‘a’, and non-words such as ‘cytpir’ were removed. Additionally, special characters and punctuations were removed. The text was formatted to lowercase letters, tokenized, and lemmatized to avoid inflected words. Common location phases such as Sim-drive and Aljunied-crescent were transformed into single words. Subsequently, data were analyzed using R software [23]. Packages including “udpipe” [24], “tm” [25], “ggplot2” [26], “wordcloud” [27], and “igraph” [28] were used for textual analysis.

### Data characteristics

Over the investigated 15 month period of the Zika outbreak (Table 1), the three government agencies posted 72 Zika-related messages on Facebook and 20 on Twitter. The public posted 947 messages relating to Zika on Facebook and 3962 on Twitter. Meanwhile, the government posts produced 236 replies and the public posts produced 2386 replies. The composition of handles for the two public data sources were further investigated (Table 2). For Facebook public posts, the majority of posts (81.6%) were published by individual users. In contrast, for public tweets, 45% of tweets were published by news media accounts and 30% by companies or communities.

**Table 1:** A summary of data sources in the study

	Data Rows	Unique Accounts
FB GOVT-POST	72	3
FB GOVT-REPLIES	236	151
FB PUBLIC-POST	947	698
FB PUBLIC-REPLIES	2386	1876
TWGOVT-TWEET	20	3
TWPUBLIC-TWEET	3962	705

*FBGOVT-POST* Facebook government posts, *FBGOVT-REPLIES* public replies to Facebook government posts, *FBPUBLIC-POST* Facebook public posts, *FBPUBLIC-REPLIES* public replies to Facebook public posts, *TWGOVT-TWEET* Twitter government tweets, *TWPUBLIC-TWEET* Twitter public tweets

**Table 2:** The composition of handles of public posts and tweets

	Facebook	Twitter
Company/Community	150	1179
Other-governments^a^	2	36
Individuals	773	953
News media	22	1794
*Total N*	*947*	*3962*

^a^Other governments refer to government agencies except for the three major health agencies MOH, NEA, and HPB in Singapore

## Results

### Timeline and discourse peaks

**Fig. 1:**
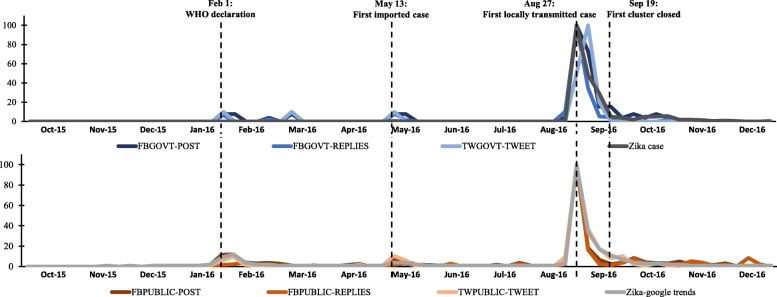
The relative frequency of discourses about Zika from 1 October 2015 to 31 December 2016, with reference to Zika confirmed cases. *[Note: To plot all figures on a common scale, figures were scaled to the highest peaks for each data source, respectively. The peak was assigned a score of 100. FBGOVT-POST = Facebook government posts; FBGOVT-REPLIES = public replies to Facebook government posts; FBPUBLIC-POST = Facebook public posts; FBPUBLIC-REPLIES = public replies to Facebook public posts; TWGOVT-TWEET = Twitter government tweets; TWPUBLIC-TWEET = Twitter public tweets]*

Figure 1 shows the timeline and discourse peaks of Zika-related social media posts. In general, the relative volume of Zika discourse mapped out on that of the Zika activity developments. The discourses of the disease began in late January around the World Health Organization’s declaration of Zika as an international health emergency. On May 13, the disease discourses demonstrated a second peak corresponding to the confirmation of the first imported Zika case in Singapore. On the week after August 27 when the first locally transmitted case was verified and the Zika outbreak was declared, the overall discourse outreach reached its highest peaks. The discourses died down quickly before the first Zika cluster was closed on September 19.

Nevertheless, there are also differences among data sources. First, the volume of Zika information on Twitter by governments reached its peak on the second week after the outbreak announcement. This is because the government updated press releases about the Zika cases via Twitter daily for over 2 weeks after the announcement. Second, the volume of public discourse on Facebook and that of the replies reached their peaks at the first week of the outbreak and died down significantly the week after. Comparing between the public discourse on Facebook and on Twitter, the former reduced more quickly than the latter.

**Fig. 2:**
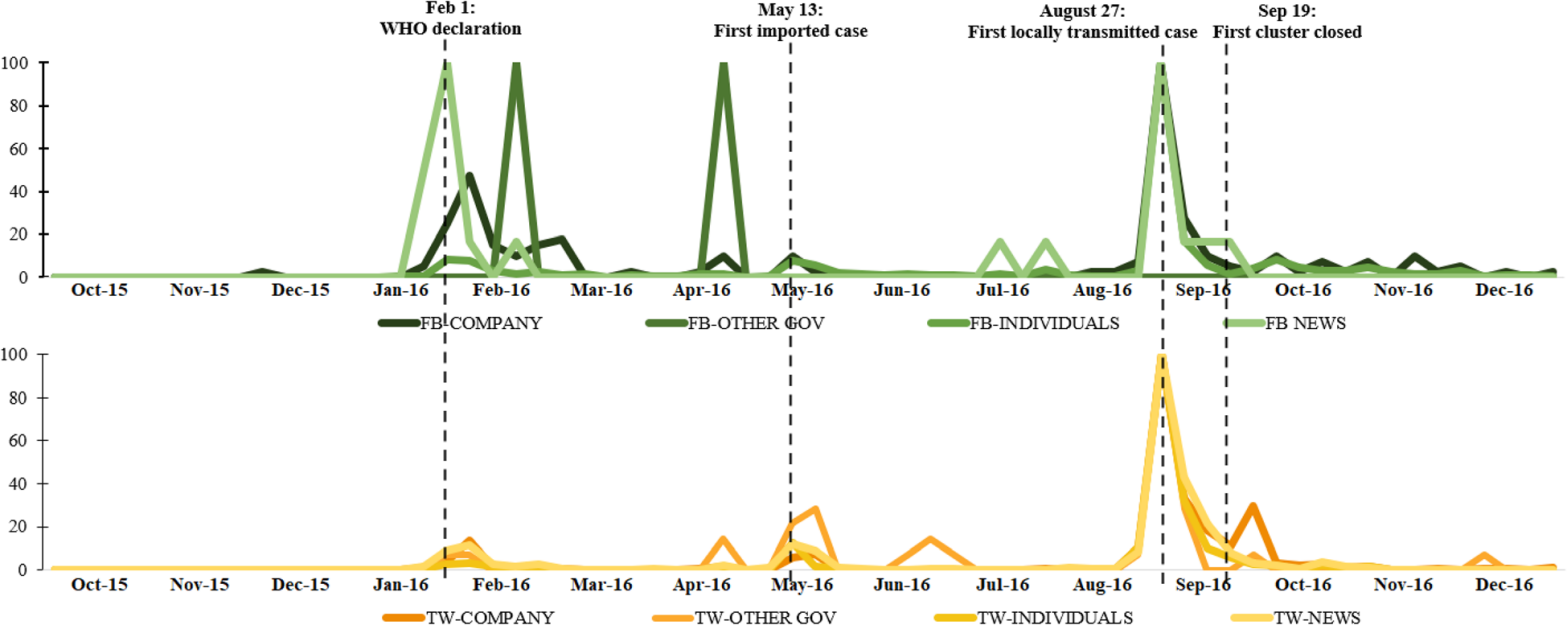
The relative frequency of discourses about Zika from 1 October 2015 to 31 December 2016 by different social media handles. *[Note: To plot all figures on a common scale, figures were scaled to the highest peaks for each data source, respectively. The peak was assigned a score of 100. FB = Facebook; TW = Twitter. FB/TW-COMPANY = Facebook posts/Tweets published by companies or communities; FB/TW-OTHER GOV = Facebook posts/Tweets published by other governments except the MOH, NEA, and HPB; FB/TW-INDIVIDUALS = Facebook posts/tweets published by individual users; FB/TW-NEWS = Facebook posts/tweets published by news agencies]*

To further understand the temporal patterns of public discourses on Zika, we plotted the temporal patterns for different types of handles on Facebook and Twitter (Fig. 2). Discourses by individual users from both Facebook and Twitter reached their peaks during the first week of the outbreak and died down significantly in the later weeks. In contrast, discourses by news accounts remained active after 2 to 4 weeks of the outbreak declaration.

In addition, to ascertain whether public discourses on social media reflect the overall interest in Zika in terms of internet search volume, the public discourses and the Internet search data from Google Trends were compared (Fig. 1). Results showed that though public discourses on both Facebook and Twitter generally mirrored the Google Trends search data, Twitter discourses best matched the Google Trends data.

**Fig. 3:**
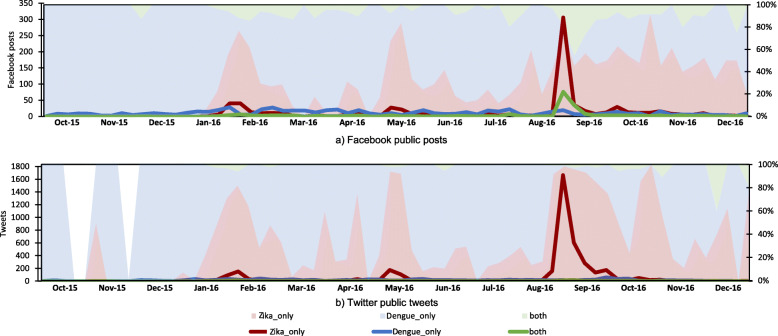
Comparison between narratives of Zika-only, dengue-only, and both, on Facebook (the upper panel) and Twitter (the lower panel)

To further understand the discourse peaks of Zika outbreak within the investigated timeline, we compared between Zika and dengue discourses (Fig. 3). Dengue is a constant disease threat to Singapore. It shares the same vector and exhibits similar symptoms (e.g., fever and rash) to Zika. Unlike the absolute frequency of discourses, the relative frequency of Zika to dengue discourses demonstrated a fourth major peak in the timeline on both Facebook and Twitter. The fourth peak occurred at epi-week 43 (i.e., October 23 to 29) in 2016. A check on the text data shows that those Zika social media posts and tweets are about the evaluation of crisis responses to Zika in Singapore, given that it had been 2 months since the outbreak was declared. In addition, there is a contrast between Facebook and Twitter that discourses on Facebook links Zika to dengue more often than those on Twitter. Overall, the timeline of Zika discourses suggests that the discourses mirrored important developments of Zika activities in Singapore.

### Dominant terms

**Fig. 4:**
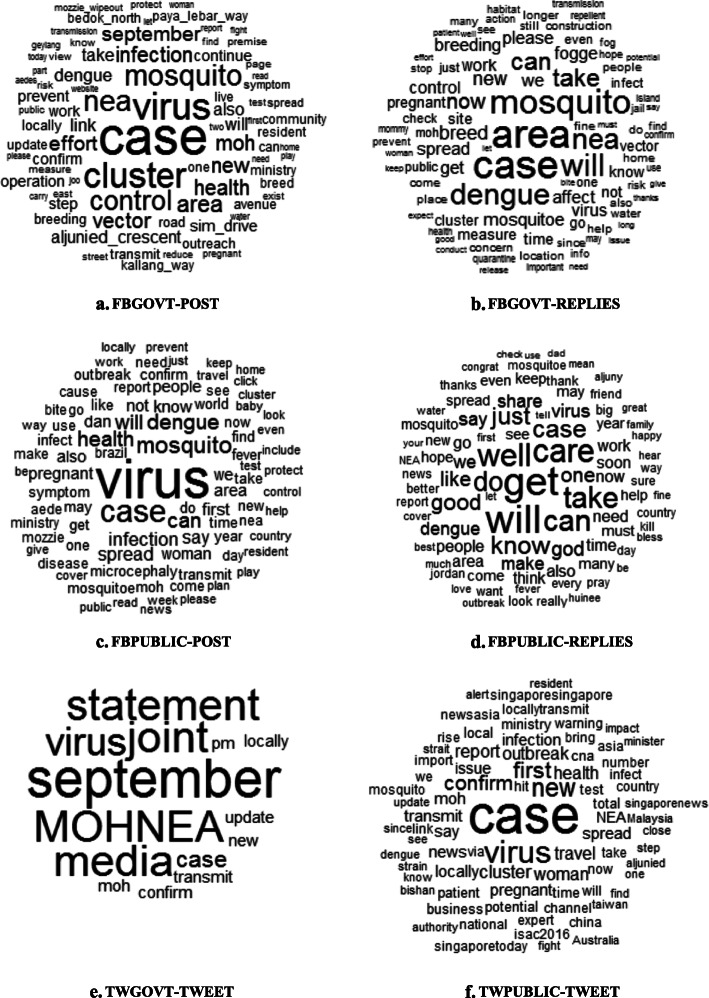
Word clouds of the most frequently used words during the 2016 Zika outbreak in Singapore across data sources (a-f). *[Note: All words were lemmatized before analysis. The search word “Zika” and location “Singapore” were removed from display. The size of words indicates its relative frequency, where words in larger fonts were more frequently used]*

To understand the general content of Zika social media posts, automatic textual analysis was conducted using *R*. All Zika-related social media posts included the word Zika, given the design of the data collection process. Figure 4 (a-f) shows word cloud visualizations of the 100 most frequently used terms for each data sources (except the word cloud of government tweets, which visualized words having a frequent larger than 1), with the size of words indicating their relative frequency.

Overall, “virus” was the most common encountered word associated with the Zika narratives across data sources. This is expected given that Zika is a kind of infectious virus. “Case” was also among the top words about Zika, reflecting the general interest in the spread of the disease in the society. “Pregnant” “woman” are also regular terms across data sources. This is likely because the disease can cause lifelong risks to the newborns of pregnant women.

Although discourses share similarities between data sources, the finer content of the discourses differed greatly among them. On Facebook, posts of government agencies contained top terms such as “cluster”, “area”, “control”, and “mosquito”. This suggests that Facebook was used by government agencies as a platform for disseminating situational updates and government interventions. The replies to government posts contained similar top terms with the government posts, such as “area” and “mosquito”. Replies to government posts also includes the word “please”, suggesting there may be recommendations from the public to the government responses. This indicates a two-way communication between the government and the public on the Zika outbreak situations. Although public posts on Facebook also involved top terms such as “area” and “mosquito”, they were also related to the discussion of “dengue”, likely due to the fact it shares the same vector with the Zika disease. The public also includes discussions of symptoms, such as “microcephaly” and “fever”. Replies to the public Facebook posts includes personal well-wishes to victims (e.g., “get”-“well”-“soon”, “take”-“care”). On Twitter, tweets of government agencies contained top words such as “September”, “joint”, and “statement”, indicating that government tweets were mostly media statement from the health agencies. Public tweets tended to be more news-related since “news”, “Singaporetoday (i.e., Singapore Today)”, and “Singaporenews (i.e., Singapore News)” were common terms in the corpus. This is consistent with the result that news media accounts made up half of the handles in the Zika discussion on Twitter.

## Discussion

### Principal findings

The investigation of Facebook and Twitter discourses during the Zika outbreak in Singapore illustrated how social media discourses could converge and differ across different groups of users on different social media platforms. The data showed that public discourse and interest about the Zika outbreak generally occurred in parallel to government agencies communicating about Zika, sharing the same peaks and troughs throughout the timeline. Looking at the content of the discourses, there are a number of key findings that illuminate some critical differences with which the public and government use social media.

First, Twitter was the preferred and most dominant platform for news media outlets. According to Table 2, although Facebook was used more by individuals in the general public, Twitter was more widely used by news media accounts. Similarly, the textual analysis showed that government agencies utilized Twitter to convey mainly media statements on the outbreak. Previous studies have highlighted the importance of Twitter being a preferred platform for breaking news, with one study showing that over 85% of tweets are headline news [29, 30]. A potential reason is that Twitter’s word limit allows for news agencies to give breaking news without necessarily committing to resources to develop longer-form news articles, or that Zika-related news is disseminated in smaller chunks over the course of the day. Meanwhile, longer-form news articles might be disseminated on Facebook [30].

Second, though the temporal patterns of different discourses across the timeline of the Zika outbreak largely mirrored the disease development, they are slightly different. Public discourses, including public Facebook posts and the replies to posts, died down quickly during the second week after the outbreak announcement. In contrast, government Facebook posts, government and public tweets, and the Zika search on Google Trends remained at relative high volumes after 2 to 4 weeks of the outbreak declaration. A deeper analysis revealed that discourses by individual users died down more quickly than those by news media on both Facebook and Twitter. The temporal differences of different discourses have not been reported in previous studies.

The above findings likely suggest that there are two different components of the Zika discourses. The first component indicates public interests in Zika *discussions*, which includes data sources from posts and tweets published by individual users and the replies. Our findings indicate that public interests in Zika discussions reduced in rapidly after the initial event of the crisis. The second component indicates public interests in Zika *information*, which includes data sources from government posts, posts and tweets published by news accounts, and Zika search on Google Trends. The findings indicate that public interests in Zika information may last for some time until the situation is considerably controlled.

Third, the structure and content of Zika conversations varied across different groups of users, suggesting different perceptual focuses of the disease between governments and the general public. Government agencies tended to be more informative in their posts, with information about updates on Zika cases and their vector control efforts. From the perspective of crisis communication, these government practices can help update crisis situations while at the same time reduce uncertainty and promote assurance to the general public [31]. In contrast, besides factual information about Zika, the general public also talked about elements of care such as disease symptoms and the health risks to newborns of pregnant women (i.e., microcephaly). There is also an element of community in the public’s replies on Facebook, with well-wishes for victims and each (e.g. “get”, “well”, “take”, “care”). These topics depict the general public perception towards the Zika disease, which is consistent with previous research [10, 11].

There are several significant implications of the findings of this study. It illustrates that social media conversations can be extracted and analyzed in order to better understand public sentiment and uncover differences communication patterns between the government and the general public during a health epidemic. By projecting social media conversations to different sources, researchers will be able to examine the collective narrative that emerges on cyberspace in real-time. Such an approach can help provide greater insight into public discourse regarding health issues, thus informing government agencies better communication strategies.

Specifically, this study highlights how different social media platforms serve different functions, with Twitter being more of a platform where news agencies share short-form breaking news, and with Facebook being more of a platform for not only longer-form statements and news, but also a platform for engagement. With most of the Facebook replies by the general public involving personal messages, it suggests that Facebook should be a platform for engagement and increased communication during an epidemic, in order for governments and health organizations to engage the general public on a personal level. Echoing [32], this study found that news media are the most dominant voices on Twitter. As a result, an “indirect” approach where governmental agencies work with news agencies to release short-form health information might provide a greater reach for relevant health information to reach the public during an epidemic.

The study also sheds light on the differentiation of public interests as the disease outbreak unfolds. While both of the interests reached their peaks right after the declaration, interests in discourse died down as quick as it grew. In contrast, interests in information last for 2 to 4 weeks until the crisis situation was controlled. This differentiation has important implications on the disease surveillance and communication strategies. In terms of disease surveillance, this study found that interests in information mapped better onto the Zika activities than interests in discourse. Regarding communication strategies, such differentiation suggests that government agencies should initiate two-way communications with the public as soon as the outbreak event is declared, while at the same time they should continually update outbreak information to fulfil the public needs for information.

### Limitations

There are some limitations to this study. The first two limitations revolved around the complexity of the Facebook and Twitter platform and their dynamic nature. While the study was focused on how Singapore and Singaporeans responded to the Zika outbreak, some data collected might not be from Singapore due to the way platforms allocate geographical information to certain users. Also, the current dataset is relatively small compared to previous social media studies. Nevertheless, it should be noted that the dataset mentioned refers to the final dataset after all filters have been fulfilled. Hence, the current dataset is highly specific to the Zika discourses in Singapore. Notably, the location filter can only be determined by self-selected location tags or mentions within the posts. Thus, all of the posts from Singapore may not have been captured, which leads to a small dataset for the current study compared to previous studies. Previous studies suggests that approximately 1% of the social media posts are tagged with geolocation [12]. Though this procedure will limit our ability to gather a larger dataset, it can ensure that our analysis reflects the trends only within Singapore. Future study should try to identify a better method for data collection so that more data can be collected for analysis within a specific city area.

Furthermore, we conducted only a quantitative content analysis with our data. Though the current analysis emphasized the temporal and content differences between user groups and social media platforms of the Zika discourse rather than the Zika narratives, an in-depth qualitative analysis on Zika narratives using the Facebook comment data would strengthen the understanding of the conversations between governments and the public and among the public. Tweets and their comments should also be collected and analyzed to understand the responses to the public to the Zika news in future research. Overall, future study should take a step more into the analysis of Zika narratives based on our current analysis.

## Conclusions

With the proliferation of the Internet, social media has been increasingly used as a platform for communicating health-related information. The study demonstrates the content and structural differences between user groups and social media platforms in social media conversations during the Zika outbreak. This suggests that researchers should examine the collective discourses that emerge from different data sources to gain better insights into the communication strategies health organizations and governments can utilize during a health epidemic.

## Data Availability

The datasets used and/or analyzed during the current study are available from the corresponding author on reasonable request.

## Abbreviations

WHO: the World Health Organization
MOH: Ministry of Health
NEA: National Environment Agency
HPB: Health Promotion Board
